# Better Than Its Reputation? Gossip and the Reasons Why We and Individuals With “Dark” Personalities Talk About Others

**DOI:** 10.3389/fpsyg.2019.01162

**Published:** 2019-05-29

**Authors:** Freda-Marie Hartung, Constanze Krohn, Marie Pirschtat

**Affiliations:** Faculty of Communication and Environment, Rhine-Waal University of Applied Sciences, Kamp-Lintfort, Germany

**Keywords:** gossip, gossip motives, situation, dark triad, narcissism, Machiavellianism, psychopathy

## Abstract

Gossip is an ubiquitous phenomenon. Hearing information about others serves important social functions such as learning without direct interaction and observation. Despite important social functions gossip has a rather negative reputation. Therefore, the present online study focuses on the reasons why people gossip and how these reasons are related to personality (i.e., dark triad) and situational settings. Six distinct motives were identified that underlie gossip behavior: information validation, information gathering, relationship building, protection, social enjoyment, and negative influence. The most important motive was validating information about the gossip target followed by the motive to acquire new information about the gossip target. The least important motive was harming the gossip target. The motivational pattern was highly similar between private and work context. Interestingly, the importance of motives mainly depends on the gossiper's narcissism both in work and in private settings. The findings suggest that the negative reputation of gossip is not justified. In fact, even “dark” personalities appear to use gossip to tune their picture of other humans and themselves and not to harm others.

## Introduction

Eavesdropping in public settings reveals that people devote a substantial part of their conversations to gossip (e.g., Levin and Arluke, 1985; Dunbar et al., 1997). Accordingly, important social functions have been postulated for gossip in science (e.g., Foster, 2004). Despite these important functions, gossip has a rather bad reputation since it is perceived as inherently malicious harming people and society (e.g., Farley, 2011; Hartung and Renner, 2013; Peters and Kashima, 2013). Whether behavior can be judged as good or bad depends, at least in part, on the intention of the individuals engaging in that behavior. Therefore, the present study aims to examine whether the bad reputation of gossip is justified by examining reasons to gossip. In addition, we examine the reasons of individuals scoring high on the dark triad personality traits (i.e., narcissism, Machiavellianism, psychopathy) as they are known to ignore commonly accepted norms and to act selfishly (e.g., O‘Boyle et al., 2012; Muris et al., 2017). More specifically, we investigate whether individuals scoring high on the dark triad personality traits are more ready to use gossip in order to harm others and to serve themselves, thereby, contributing to the negative reputation of gossip.

Gossip refers to the exchange of information about characteristics and behaviors of an absent person (Dunbar, 2004b; Foster, 2004; Peters and Kashima, 2015). From an anthropological perspective, it has been argued that human language primarily evolved to exchange social information in order to deal with complex social situations (Dunbar, 1998, 2004a; Barrett et al., 2002), and that we, therefore, preferentially attend to social information (e.g., Mesoudi et al., 2006). Accordingly, two thirds of adult conversations in public settings involve gossip (e.g., Levin and Arluke, 1985; Dunbar et al., 1997). Experimental evidence is in line with that notion (e.g., Mesoudi et al., 2006). In general, gossip appears to be a widely spread and almost inevitable phenomenon.

As a result, important social functions have been postulated for gossip in anthropological and psychological science (e.g., Suls, 1977; Baumeister et al., 2004; Dunbar, 2004a,b; Foster, 2004; Hartung and Renner, 2013). First, gossip is an efficient means of gathering and disseminating information (Foster, 2004). The exchanged information enables individuals to get a map of their social environment and their position within that social environment (Suls, 1977; Baumeister et al., 2004; Foster, 2004; De Backer et al., 2007; Sommerfeld et al., 2007; Martinescu et al., 2014). Baumeister et al. (2004), for instance, understand gossip as an extension of observational learning. People learn about the complex social and cultural life by hearing about the success and misadventures of others. It appears that we do not learn only about extraordinary experience made by others but also about more trivial things such as dressing style (De Backer et al., 2007). Thus, exchanging information about others enables us to learn without direct interaction and observation.

Secondly, Dunbar (1998, 2004a) and Mesoudi et al. (2006) argues in his *social gossip theory of language* that human language evolved in order to keep track of complex social networks and to ensure the cohesion in large social groups. More specifically, it has been suggested and empirically shown that, at the dyadic level, sharing gossip is associated with friendship (Grosser et al., 2010; Watson, 2011; Ellwardt et al., 2012b) and even leads to the development of friendships (Ellwardt et al., 2012b; see also Bosson et al., 2006). In addition, it has been suggested that, at the group level, gossip leads to group specific knowledge, norms, and trust, in turn supporting group cohesion and bonding (e.g., Dunbar, 2004b; Foster, 2004; Peters et al., 2017). Thus, sharing information about others is a way to build and maintain relationships and networks.

Thirdly, a growing number of researchers assume that gossip serves as an informal policing device for controlling free riders and social cheats (Dunbar, 2004b; Keltner et al., 2008; Feinberg et al., 2012). Faced with the concern that information about negative behavior runs through the grapevine and may consequently lead to the loss of reputation or even social exclusion, it prevents people from acting against social norms and the good of the group (Piazza and Bering, 2008; Beersma and Van Kleef, 2011; Feinberg et al., 2014; Wu et al., 2016). Thus, gossip keeps people from acting against the good of the group and fosters cooperation.

Finally, it has been suggested that gossip has an entertainment function providing recreational value and considerable stimulation for very little costs (Foster, 2004; Peng et al., 2015). Taken together, research has postulated and empirically shown that the exchange of information about absent third persons serves several important functions in a social environment.

However, despite its important social functions, gossip has a rather negative reputation (Farley, 2011; Hartung and Renner, 2013; Peters and Kashima, 2013). For instance, asking individuals to rate their tendency to gossip, they rate themselves to be less gossipy than an average peer of the same sex, suggesting that gossiping is perceived rather negatively (Hartung and Renner, 2013). Also, frequent gossipers are perceived as less likable and less popular than people gossiping less frequently (Farley, 2011; Ellwardt et al., 2012b). Supporting the bad reputation, some researchers suggest that gossip is a covert form of aggression (i.e., non-confrontational) especially used by women (e.g., McAndrew, 2014). Thus, the positive “social function view” is not mirrored in the reputation of gossip and gossipers.

Thus, evaluating gossip as a rather positive or negative behavior is not as easy as it may appear at first sight. Focusing on the social functions, that can be understood as not necessarily intended social consequences of gossip behavior, research clearly paints a positive picture of gossip. However, one might also evaluate gossip with respect to other dimensions such as positivity or negativity of the transmitted information or the intention of the gossiper (Eckhaus and Ben-Hador, 2018). Focusing on one of these dimensions of gossip might change the evaluation and emphasize the negative reputation of gossip. And indeed, research has shown that people give consideration to the fact that gossip differs and also gossipers differ from each other (Farley, 2011; Beersma and Van Kleef, 2012; Peters and Kashima, 2015). Empirical findings have shown that people take the presumed motivation of a gossiper into account when judging the morality of the respective gossiper, for instance (Beersma and Van Kleef, 2012). Thus, even though people disapprove of gossip in general, they consider the reasons people might have to gossip.

Hence, to evaluate whether a certain behavior is good or bad, the underlying reasons or the intentions should be taken into account. Curiously, very few research exists on simply asking people about the reasons why they gossip (Beersma and Van Kleef, 2012). In their study, Beersma and Van Kleef (2012) distinguished four different reasons to gossip, namely *information gathering and validation, social enjoyment, negative influence*, and *group protection*. This means, people instigate gossip to gather information and compare their ideas about others, to enjoy themselves, to spread negative information about a third person, and/or to protect the person they are talking with. The study provides initial evidence that people primarily gossip to gain information about other people and not to harm others (Beersma and Van Kleef, 2012). Thus, when focusing on gossipers' intentions, a rather positive picture of gossip is painted.

Another way to explore whether the reputation of gossip is justified is to examine the gossip reasons of individuals scoring high on narcissism, Machiavellianism and psychopathy. These three traits are summarized under the umbrella term *dark triad* and gained considerable attention in the past years (Jones and Paulhus, 2011; O‘Boyle et al., 2012; Furnham et al., 2013; Jones and Figueredo, 2013; Lee et al., 2013; Book et al., 2015; Muris et al., 2017). It has been shown that the three traits are overlapping, but are nevertheless distinct concepts (e.g., Furnham et al., 2013; Lee et al., 2013; but see also Muris et al., 2017). As the common core the tendency to deceive, manipulate, and exploit others for one's own benefit has been suggested (Lee et al., 2013; see also Jones and Figueredo, 2013). Conversations about absent third parties appear to be an apparent method to do exactly that. Thus, if individuals with “dark” personalities regularly use gossip to spread negative information and harm others that would surely contribute to the negative reputation of gossip. However, if even individuals with “dark” personalities rarely use gossip with the intention to harm others, the positive aspects of gossip would be underlined.

Research has shown that the dark triad personality traits are related to a variety of negative social and non-social outcomes (e.g., Baughman et al., 2012; O‘Boyle et al., 2012; Wisse and Sleebos, 2016; Muris et al., 2017; Deutchman and Sullivan, 2018). For instance, individuals scoring higher on the dark triad traits show a higher tendency to tell lies and to cheat than individuals scoring lower on these traits (Nathanson et al., 2006; Williams et al., 2010; Baughman et al., 2014; Jonason et al., 2014; Roeser et al., 2016; Muris et al., 2017). In addition, individuals scoring higher on the dark triad value themselves over the others (Jonason et al., 2015), are less concerned with others' welfare (Djeriouat and Trémolière, 2014; Jonason et al., 2015; Noser et al., 2015) and with fairness (Jonason et al., 2015). Taken together, these studies and reviews illustrate that individuals scoring higher on the dark triad personality traits are willing to dismiss commonly accepted social norms and harm others for their own good.

Therefore, it is plausible to assume that individuals scoring higher on the dark traits are also more ready to use gossip for their own sake without caring about potentially negative effects for others. More specifically, it is easy to imagine that individuals scoring higher on the dark triad readily use gossip to negatively influence another person's reputation (i.e., potential competitor or rival) to push through self-beneficial agendas. In line with that notion, women scoring high on the dark triad traits use gossip—among other strategies—to derogate competitors (Carter et al., 2015). Additionally, as people with dark personalities are not concerned with others' welfare, they probably use gossip less often to protect other individuals or their group from harm (but see Lyons and Hughes, 2015). In a similar vein, the dark side of personality probably has a high impact on gossip motives that serve individual purposes. For instance, people scoring high on the dark triad traits report to have a strong desire for power, control, and dominance (e.g., Jonason et al., 2010; Lee et al., 2013; Semenyna and Honey, 2015). Gaining social information and knowledge about people surrounding us provides us with a sense of control and advantage over others (e.g., Swann et al., 1981; Fiske, 2004). Therefore, simply gathering and validating social information might be another salient reason for dark personalities to gossip.

Taken together, the present online study focuses on the reasons why people engage in conversations about absent third parties. The aims of the present study are 2-fold. First, we aim to examine the reasons for people to engage in gossip, replicating the study of Beersma and Van Kleef (2012). To do so, we translated the *Motives to Gossip Questionnaire* into German. In addition, we extended the questionnaire by widening the number of possible reasons including gossiping in order to foster relationship building and to gather social information. Second, to examine whether the bad reputation of gossip is justified or not, we explore the role of the “dark” personality traits in gossip motivation. One might assume that individuals scoring higher on narcissism, Machiavellianism, and psychopathy are more likely unconcerned with moral considerations and driven by selfish reasons when engaging in gossip, consequently, contributing to the negative reputation of gossip. However, we have no specific hypotheses concerning the single dark triad traits.

The *Motives to Gossip Questionnaire* asks participants to rate their reasons for gossip in a specific situation. In order to explore to what extent gossip motives can be generalized across situations, two different situations were incorporated in the study (i.e., private as well as workplace situations). Based on the work of Mischel (1977), researchers differentiate between strong situations with normative expectations and clear roles that constrain behavior, and weak situations which do not provide normative expectations, and, therefore, allow for more freedom in behavior and the expression of personality. Mischel (1977) argued that behavior in strong situations is based on situational circumstances rather than on the individual's personality. In the workplace, people have to follow rules and adjust their behavior to fulfill or support organizational objectives. Here we can assume rather strong situations. In private situations on the other hand, people are mostly unrestricted and have to comply with fewer norms or rules. Also, it is likely that work and private setting differ on a competitiveness-cooperativeness dimension. A competitive situation might elicit motives that serve the individual more easily and hazards negative consequences for others. Taken together, we assume that the work context reflects a rather strong (i.e., clear normative expectations) and competitive situation; and the private context reflects a rather weak and more cooperative situation. Consequently, we explore whether motives show differential importance between these two situations and whether the dark triad traits show differential relationships to gossip reasons across situations (see also Beersma and Van Kleef, 2012).

## Method

### Procedure

Participants were invited via e-mail to fill in an online questionnaire about communication at work. In total, 40 employees from different companies in Germany were addressed. For snowball sampling they were asked to distribute the link to colleagues and other employees. Participants were informed about the study content, that they were free to withdraw at any time without giving any reason, and that the data collection and analysis were anonymized. The study conforms with the Declaration of Helsinki and the ethics guidelines of the German Psychological Society. In accordance with the national and institutional guidelines, ethical approval was not required for this study. The questionnaire was conducted with the informed consent of each subject. Informed consent was provided by ticking a box indicating comprehension of instruction and agreement that their data is used for scientific purposes. Approximately 15–20 min were required to answer the questionnaire. For every questionnaire that was filled in completely 50 Cent were donated to the UNO-Flüchtlingshilfe (UN refugee relief).

### Participants

In total, 134 participants (*n* = 79 women, 59%) with a mean age of 35.25 years (*SD* = 13.10, range = 21–78 years) were recruited for the study. The majority were employed (employed, *n* = 85), *n* = 44 were students, *n* = 3 were retired, *n* = 1 were in apprenticeship, and *n* = 1 was unemployed. In total, 100% of the students and the unemployed participant reported to have work experience.

### Measures

#### Motives to Gossip

To measure reasons to gossip the English version of *Motives to Gossip Questionnaire* (Beersma and Van Kleef, 2012) was translated into German using the parallel blind technique (Behling and Law, 2000). That is, four bilingual individuals (German native speakers) translated the questionnaire independently and subsequently reached an agreement on the final version. In addition, that final version was presented to two bilingual individuals (English native speakers) to review the final version. The *Motives to Gossip Questionnaire* contains 22 items tapping into four different motives, namely the *information gathering and validation* motive (nine items), the *social enjoyment* motive (five items), the *negative influence* motive (five items), and the *group protection* motive (three items).

Some modifications were made to the original version. To consider a relationship building motive of gossip, three respective items were generated. To distinguish between information gathering and information validation, three new items were generated to capture *information gathering*; three items of the *information gathering and validation* subscale were chosen to represent *information validation*. Moreover, to create a concise measure three items were chosen from the respective subscale to represent the *social enjoyment* motive and the *negative influence* motive. The three items were chosen based on consideration about the content and wording as well as on factor loadings obtained through a pre-study (*N* = 45).

**Figure 1:**
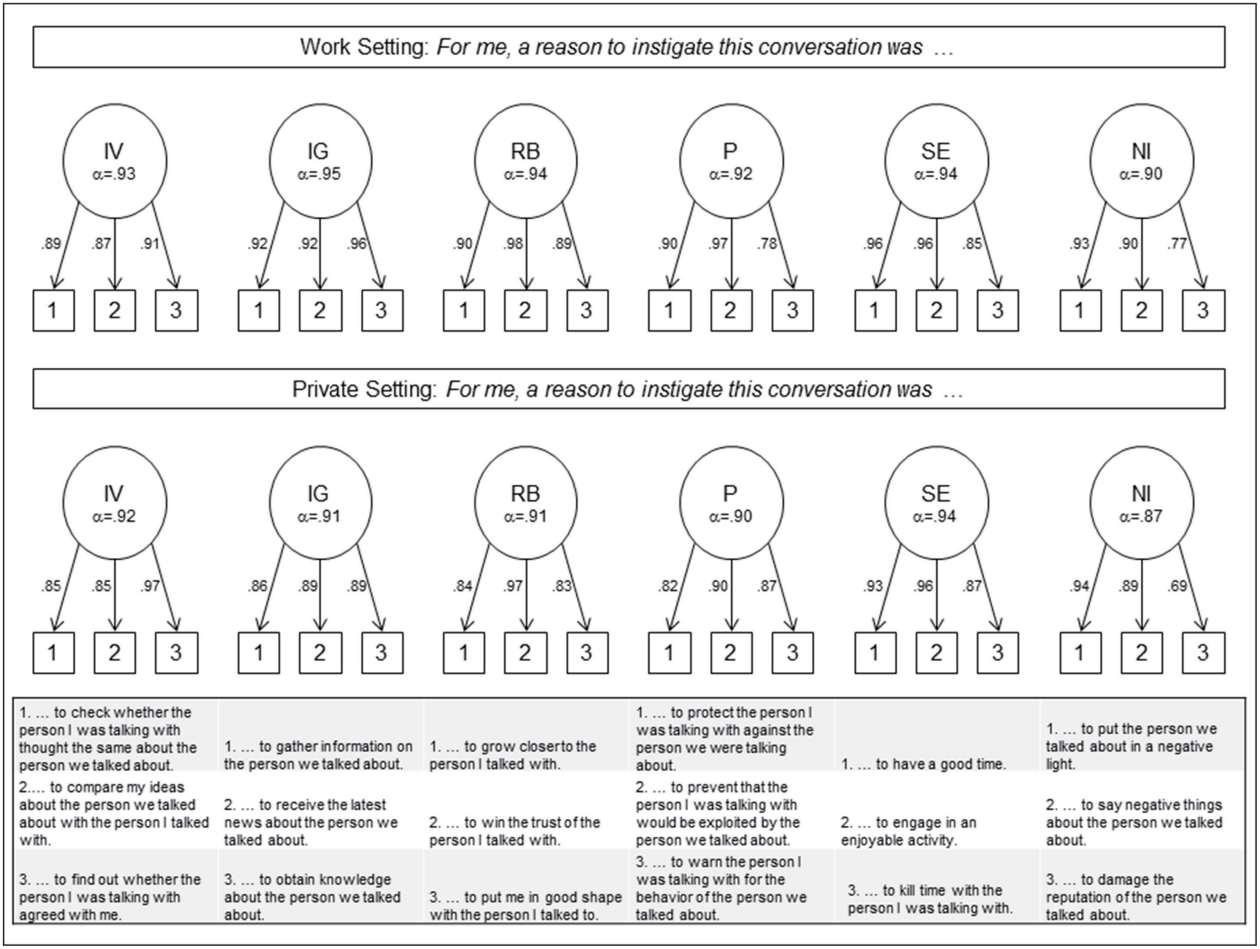
Factor loadings of the items of the *Motives to Gossip Questionnaire*—*Revised* (confirmatory factor analysis) separately displayed for work and private setting (*N* = 134). IV, Information Validation; IG, Information Gathering; RB, Relationship Building; P, Protection; SE, Social Enjoyment; NI, Negative Influence. Latent factors are allowed to correlate. However, correlations are not displayed due to clarity reasons.

Taken together, the preliminary scale consists of 18 items tapping into six different motives, namely *information gathering* (IG), *information validation* (IV), *relationship building* (RB), *protection* (P), *social enjoyment* (SE), and *negative influence* (NI; see Figure 1). Ratings were provided on a 7-point scale ranging from 1 (*completely disagree*) to 7 (*completely agree*).

Consistent with Beersma and Van Kleef (2012), we asked the participants to think about a past situation when they had a conversation with someone about an absent person. We asked them to think about the reasons they had for that conversation and to answer the *Motives to Gossip Questionnaire-Revised* accordingly. Unlike Beersma and Van Kleef (2012), we asked the participants to think about a situation in a work setting as well as in a private context. Thus, participants filled in the questionnaire twice.

To ensure the internal validity, confirmatory factor analyses (CFAs) using AMOS (version 24.0.0) were conducted for private and work setting separately (see Figure 1). The models tested the hypothesized six-factorial model with the six scales as correlated first-order factors with paths leading to the three items hypothesized to comprise that factor (see Figure 1). The chi-square statistic was significant for the work setting (χ^2^_(120)_ = 190.92, *p* < 0.001) but not for the private setting χ^2^_(120)_ = 131.37, *p* = 0.22). For both settings, the comparative fit index (work setting: CFI = 0.97; private setting: CFI = 0.99) and the root mean square error of approximation [work setting: RMSEA = 0.07 [90% CI:0.05, 0.08]; private setting: RMSEA = 0.03 [90% CI:0.00, 0.05]] were in the acceptable range. The standardized factor loadings for the six-factor model are presented separately in Figure 1 for both settings. All factor loadings were significant (*p* < 0.001). The inter-factor correlations varied in between *r* = 0.10 and 0.61 for the work setting and between *r* = −0.17 and 0.43 for the private setting. Due to clarity reasons the inter-factor correlations are not displayed in Figure 1. They are, however, highly similar to bivariate correlations displayed in Table 1. Internal consistency was highly satisfactory for all subscales (see Figure 1) and, if applicable, comparable to those of Beersma and Van Kleef (2012). The German version of the extended *Motives to Gossip Questionnaire* is displayed in the Appendix.

**Table 1:** Correlations between all scales (*N* = 134).

			**Gossip motives**											**Dark triad**		
			**Work setting**					**Private setting**								
			**IG**	**RB**	**P**	**SE**	**NI**	**IV**	**IG**	**RB**	**P**	**SE**	**NI**	**N**	**M**	**PS**
Gossip motives	Work setting	Information validation	0.57^**^	0.44^**^	0.16	0.33^**^	0.12	0.67^**^	0.28^**^	0.29^**^	0.09	0.08	0.03	0.32^***^	0.17^*^	0.08
		Information gathering		0.44^**^	0.14	0.46^**^	0.14	0.43^**^	0.49^**^	0.35^**^	0.13	0.31^**^	0.14	0.35^***^	0.30^***^	0.05
		Relationship building			0.37^**^	0.50^**^	0.39^**^	0.42^**^	0.33^**^	0.73^**^	0.16	0.36^**^	0.35^**^	0.48^***^	0.36^***^	0.21^**^
		Protection				0.09	0.24^**^	0.18^*^	0.13	0.29^**^	0.50^**^	0.10	0.15	0.29^***^	0.28^***^	0.11
		Social enjoyment					0.35^**^	0.21^*^	0.29^**^	0.47^**^	0.08	0.62^**^	0.41^**^	0.28^***^	0.23^**^	0.16
		Negative influence						0.13	0.19^*^	0.30^**^	0.14	0.31^**^	0.63^**^	0.33^***^	0.42^***^	0.29^***^
	Private setting	Information validation							0.31^**^	0.33^**^	0.07	0.08	0.11	0.29^***^	0.11	0.01
		Information gathering								0.40^**^	0.12	0.34^**^	0.24^**^	0.16	0.22^**^	0.08
		Relationship building									0.17^*^	0.38^**^	0.28^**^	0.34^***^	0.26^**^	0.17^*^
		Protection										−0.16	0.22^*^	0.09	0.10	0.05
		Social enjoyment											0.30^**^	0.22^**^	0.16	0.20^*^
		Negative influence												0.35^***^	0.32^***^	0.23^**^
Dark triad		Narcissism													0.61^***^	0.34^***^
		Machiavellianism														0.49^***^

*N, Narcissism; M, Machiavellianism; PS, Psychopathy; IV, Information Validation; IG, Information Gathering; RB, Relationship Building; P, Protection; SE, Social Enjoyment; NI, Negative Influence*;

*** *p < 0.001*;

** *p < 0.01*;

* *p < 0.05*.

#### Dark Triad

The dark triad personality traits were measured using the German version of the Dirty Dozen scale (DD, German version: Küfner et al., 2014; original version: Jonason and Webster, 2010). The Dirty Dozen scale captures narcissism (e.g., “I tend to want others to admire me.”), Machiavellianism (e.g., “I tend to manipulate others to get my way.”), and psychopathy (e.g., “I tend to be callous or insensitive.”) with four items for each subscale. Ratings were provided on a 9-point rating scale ranging from 1 (*disagree strongly*) to 9 (*agree strongly*). Thus, higher values indicate higher degrees in the respective personality trait. The subscales exhibited satisfactory reliability in the present study with α = 0.84 for narcissism, α = 0.85 for Machiavellianism, and α = 0.75 for psychopathy which is comparable to previous research (Küfner et al., 2014). On average, participants had scores of *M* = 4.50 (*SD* = 1.92) on the narcissism subscale, of *M* = 3.40 (*SD* = 1.78) on the Machiavellianism subscale, and of *M* = 2.70 (*SD* = 1.66) on the psychopathy subscale. The mean values for narcissism and Machiavellianism are comparable to previous research whereas the mean value for psychopathy is slightly lower in the present study than in previous research (Küfner et al., 2014). There are no gender effects for narcissism (women *M* = 4.33, *SD* = 2.07 vs. men *M* = 4.73, *SD* = 1.68; *t*_(132)_ = −1.19, *p* = 0.24, Cohen's *d* = 0.21) and Machiavellianism (women *M* = 3.30, *SD* = 1.72 vs. men *M* = 3.54, *SD* = 1.88; *t*_(132)_ = −0.76, *p* = 0.45, Cohen's *d* = 0.13). However, men and women differ significantly with regard to psychopathy (women *M* = 2.50, *SD* = 1.58 vs. men *M* = 3.08, *SD* = 1.73; *t*_(132)_ = −2.01, *p* = 0.05, Cohen's *d* = 0.35).

Bivariate correlations between all variables are displayed in Table 1.

### Analytical Procedure

Eleven participants had missing values varying between 1.80 and 35.70%. However, only four participants had missing values between 28.60 and 35.70%. Excluding these participants from analysis did not change the results. According to standard procedures, missing values were imputed prior to forming scales using the EM method in SPSS 24 (Schafer and Graham, 2002).

To examine whether the importance of motives differ among each other and between work and private situations, a repeated 6 × 2 analysis of variance (ANOVA) was conducted with both “motives” (i.e., *information validation* vs. *information gathering* vs. *relationship building* vs. *protection* vs. *social enjoyment* vs. *negative influence*) and “situation” (i.e., private vs. work) as within factors. The repeated ANOVA was also calculated including gender as a between subject factor. However, we found neither a significant effect nor did the results change including gender. Therefore, due to parsimonious reasons, we only report the results not controlling for gender.

To examine whether the importance of motives depends on the personality of the gossiper, multiple regression analyses were conducted with the dark triad personality traits as independent variables and motives as dependent variables for both work and private situations, respectively. All regression analyses were also calculated including gender as control variable. However, we found neither a significant effect nor did the results change including gender. Due to parsimonious reasons, we only report the results not controlling for gender.

In order to get more insight into our results, we additionally conducted a Bayesian Repeated ANOVA and Bayesian Regression Analyses. The Bayesian analysis has several advantages over classical statistical inference (e.g., van de Schoot et al., 2013; Wagenmakers et al., 2018b) such as less susceptibility to small sample size (van de Schoot et al., 2013). Also, the *p*-value in classical analysis provides the information about the probability of obtaining results as least as extreme as those observed given that the null hypothesis is true; the alternative hypothesis is left unspecified (Wagenmakers et al., 2018b). In contrast, the Bayes factor (BF) provided in Bayesian analysis is comparative as it weighs the support for one model against that of another. More specifically, the BF compares two competing models: Null hypothesis and alternative hypothesis (Wagenmakers et al., 2018b). BF_10_ indicates the Bayes factor in favor of H_1_ over H_0_, that is, gives the likelihood of the data under the alternative hypothesis divided by the likelihood of the data under null hypothesis. BF_01_ indicates the Bayes factor in favor of H_0_ over H_1_, that is, gives the likelihood of the data under the null hypothesis divided by the likelihood of the data under alternative hypothesis (Nuzzo, 2017; Halter, 2018; Wagenmakers et al., 2018b). According to Wagenmakers et al. (2018a) a BF_10_ > 100 indicates extreme evidence for H_1_, a BF_10_ = 30–100 indicates very strong evidence for H_1_, a BF_10_ = 10–30 indicates strong evidence for H_1_, a BF_10_ = 3–10 indicates moderate evidence for H_1_, a BF_10_ = 1–3 signals anecdotal evidence for H_1_, BF_10_ = 1 indicates no evidence for H_1_, BF_10_ = 0.3–1 signals anecdotal evidence for H_0_, BF_10_ = 0.1–0.3 indicates moderate evidence for H_0_, BF_10_ = 0.03–0.1 signals strong evidence for H_0_, BF_10_ = 0.01–0.03 indicates very strong evidence for H_0_, and a BF_10_ < 0.01 indicates extreme evidence for H_0_. Bayesian analyses were conducted using the JASP statistic package (version 0.9.2).

## Results

### Why Are We Talking About Other People?

To test differences in motives to talk about others, a 6 × 2 repeated measures ANOVA was conducted with both “motives” (i.e., *information validation* vs. *information gathering* vs. *relationship building* vs. *protection* vs. *social enjoyment* vs. *negative influence*) and “situation” (i.e., private vs. work) as within factors.

The Mauchly test effects for sphericity yielded significant effects for “motives” (χ^2^_(14)_ = 52.73, *p* < 0.001) and for “situation^*^motives” (χ^2^_(14)_ = 58.71, *p* < 0.001). Therefore, corrected *F*-values are reported (Huynh-Feldt).

The ANOVA yielded a significant main effect for the factor “motives,” *F*(4.48, 665) = 61.54, *p* < 0.001, $\eta_{\text{P}}^{2}$ = 0.32, indicating that motives were differentially important. Bonferroni adjusted *post-hoc* analysis revealed that *information validation* (*M* = 4.68, *SD* = 1.59), *information gathering* (*M* = 3.92, *SD* = 1.65) as well as *negative influence* (*M* = 2.14, *SD* = 1.26) differed significantly from all other motives (*p*s < 0.001, respectively). In contrast, *relationship building* (*M* = 3.16, *SD* = 1.70), *protection* (*M* = 3.10, *SD* = 1.58), and *social enjoyment* (*M* = 2.83, *SD* = 1.64) did not differ significantly from each other (*p*s = 0.35–1.0).

No significant main effect for the factor “situation” was yielded indicating that the importance of motives was comparable for private and work-related situations, *F*_(1, 133)_ = 2.94, *p* = 0.09, $\eta_{\text{P}}^{2}$ = 0.02.

**Figure 2:**
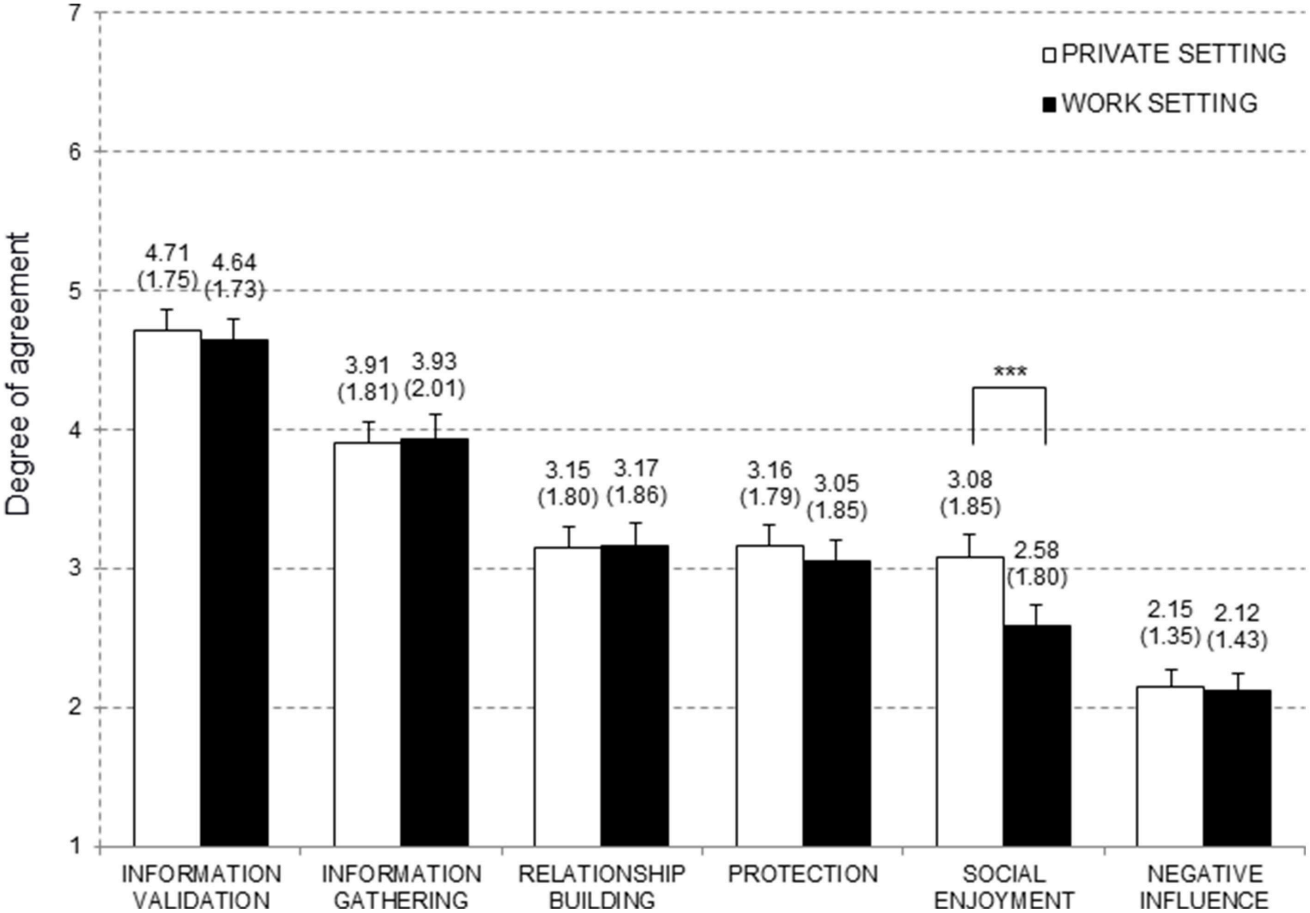
Means, standard deviation (in parenthesis), and error bars for the different motives displayed separately for work and private setting (*N* = 134).

In addition, the ANOVA yielded a significant interaction effect when using the Huynh-Feldt corrected statistics, *F*_(4.30, 665)_ = 2.29, *p* = 0.05, $\eta_{\text{P}}^{2}$ = 0.02. Bonferroni adjusted *post-hoc* analysis showed that *social enjoyment* was a more important motive in private situations than in work-related situations (private *M* = 3.08, *SD* = 1.84 vs. work *M* = 2.58, *SD* = 1.80; *p* < 0.001). All other motives were equally important in private and work settings (*p* > 0.49; see Figure 2). However, when using the more conservative Greenhouse-Geisser corrected statistics, the ANOVA yielded no significant interaction effect, *F*_(4.15, 665)_ = 2.29, *p* = 0.06, $\eta_{\text{P}}^{2}$ = 0.02.

According to Bayesian repeated ANOVA, the model containing the two main effects and the interaction effect received overwhelming support from the data with a BF_10_ = 6.33 × 10^79^. According to Wagenmakers et al. (2018a), a BF > 100 shows extreme evidence for H_1_. However, the model that receives most support was the model containing the “motives” factor only (BF_10_ = 4.48 × 10^82^). This indicates that that the data are 4.48 × 10^82^ times more likely under the model that assumes differences between motives than under the model that assumes no such differences. Also, the values of BF_inclusion_ for “situation” (BF_inclusion_ = 0.11), “motives” (BF_inclusion_ = 3.00 × 10^15^), and “situation^*^motives” (BF_inclusion_ = 4.84 × 10^−3^) show that the “situation” factor and the interaction only receive weak support. In contrast, the “motives” factor receives extreme support.

Taken together, both the classical repeated ANOVA and the Bayesian repeated ANOVA show that the “motives” factor is most meaningful in explaining the data.

### Personality and Motives to Talk About Other People

To examine whether the importance of motives depends on the gossiper's personality, multiple regression analyses were conducted for both work and private situations. The dark triad personality traits were entered as independent variables and motives as dependent variables. The results are displayed in Table 2. Noticeably, the results revealed that the importance of motives mainly depends on the gossiper's narcissism both in work settings and in private settings.

**Table 2:** Regression analysis with motives as dependent variables and dark triad personality traits as independent variables (*N* = 134).

	**IV**				**IG**				**RB**				**P**				**SE**				**NI**			
	***B(SE)***	**β**	***t***	***p***	***B(SE)***	**β**	***t***	***p***	***B(SE)***	**β**	***t***	***p***	***B(SE)***	**β**	***t***	***p***	***B(SE)***	**β**	***t***	***p***	***B(SE)***	**β**	***t***	***p***
**WORK SETTING**																								
Intercept	3.44(0.39)		8.91	<0.001	2.34 (0.44)		5.36	<0.001	0.99 (0.38)		2.58	0.01	1.72 (0.41)		4.17	<0.001	1.31 (0.41)		3.21	0.002	0.70 (0.31)		2.28	0.02
N	0.31(0.10)	0.35	3.31	0.001	0.30(0.11)	0.29	2.77	0.006	0.40 (0.09)	0.41	4.24	<0.001	0.19 (0.10)	0.20	1.87	0.06	0.20 (0.10)	0.22	2.01	0.05	0.08 (0.08)	0.11	1.08	0.28
M	−0.02(0.11)	−0.02	−0.20	0.84	0.23 (0.12)	0.20	1.84	0.07	0.11 (0.11)	0.11	1.01	0.31	0.19 (0.12)	0.19	1.65	0.10	0.08 (0.11)	0.08	0.69	0.49	0.24 (0.09)	0.30	2.82	0.006
PS	−0.05(0.10)	−0.05	−0.48	0.63	-0.19 (0.11)	−0.16	−1.67	0.10	0.00 (0.10)	0.00	0.05	0.96	-0.07 (0.11)	−0.06	−0.63	0.53	0.04 (0.11)	0.04	0.36	0.72	0.09 (0.08)	0.10	1.11	0.27
*R* (*R*^2^)	0.32 (0.10)				0.39 (0.15)				0.49 (0.24)				0.32 (0.10)				0.29 (0.08)				0.44 (0.19)			
*F, p*	*F*_(3, 130)_ = 5.00, *p* = 0.003				*F*_(3, 130)_ = 7.67, *p* < 0.001				*F*_(3, 130)_ = 13.38, *p* < 0.001				*F*(3,130) = 4.91, *p* = 0.003				*F*(3,130) = 3.89, *p* = 0.01				*F*(3,130) = 10.21, *p* < 0.001			
A: BF_10_	8.92				209.20				122473.60				7.95				2.30				3804.14			
B: BF_10_	123.92 (only narcissism)				491.71 (only narcissism)				1.92x10^6^ (only narcissism)				36.39 (only narcissism)				23.06 (only narcissism)				24451.65 (only machia.)			
**PRIVATE SETTING**																								
Intercept	3.72(0.39)		9.48	<0.001	3.10(0.42)		7.47	<0.001	1.63(0.40)		4.11	<0.001	2.72(0.42)		6.48	<0.001	1.96(0.42)		4.64	<0.001	0.90(0.30)		3.06	0.003
N	0.34(0.10)	0.37	3.48	0.001	0.04(0.10)	0.04	0.40	0.69	0.27(0.10)	0.28	2.72	0.007	0.05(0.10)	0.05	0.47	0.64	0.17(0.10)	0.18	1.63	0.11	0.16(0.07)	0.23	2.21	0.03
M	−0.07(0.11)	−0.07	−0.62	0.54	0.22(0.12)	0.22	1.88	0.06	0.08(0.11)	0.08	0.67	0.50	0.07(0.12)	0.07	0.62	0.54	-0.02(0.12)	−0.02	−0.17	0.87	0.11(0.08)	0.15	1.38	0.17
PS	−0.10(0.10)	−0.10	−1.01	0.32	-0.05(0.11)	−0.04	−0.42	0.67	0.02(0.10)	0.02	0.23	0.82	-0.01(0.11)	−0.01	−0.10	0.92	0.16(0.11)	0.14	1.44	0.15	0.05(0.08)	0.07	0.70	0.48
*R* (*R*^2^)	0.31 (0.10)				0.23 (0.05)				0.35 (0.12)				0.11 (0.01)				0.25 (0.06)				0.38 (0.14)			
*F, p*	*F*(3,130) = 4.69, *p* = 0.004				*F*(3,130) = 2.37, *p* = 0.07				*F*(3,130) = 5.87, *p* = 0.001				*F*(3,130) = 0.51, *p* = 0.68				*F*(3,130) = 2.95, *p* = 0.04				*F*(3,130) = 7.21, *p* < 0.001			
A: BF_10_	6.09				0.35				25.36				0.03				0.73				123.43			
B: BF_10_	36.69 (only narcissism)				4.10 (only Machiavellianism)				323.46 (only narcissism)				0.34 (only Machiavellianism)				3.64 (only narcissism)				455.18 (only narcissism)			

*N, Narcissism; M, Machiavellianism; PS, Psychopathy; IV, Information Validation; IG, Information Gathering; RB, Relationship Building; P, Protection; SE, Social Enjoyment; NI, Negative Influence; A: BF_10_ = probability of the alternative hypothesis compared to the null hypothesis when all three personality traits are included in the model. B: BF_10_ = probability of the alternative hypothesis compared to the null hypothesis when the model is considered under which the data are most likely; BF_10_ > 100 = extreme evidence for H_1_, BF_10_ 30–100 = very strong evidence for H_1_, BF_10_ 10-30 = strong evidence for H_1_, BF_10_ 3–10 = moderate evidence for H_1_, BF_10_ 1–3 = anecdotal evidence for H_1_, BF_10_ 1 = no evidence for H_1_, BF_10_ 1/3–1 = anecdotal evidence for H_0_, BF_10_ 1/10–1/3 = moderate evidence for H_0_, BF_10_ 1/30–1/10 = strong evidence for H_0_, BF_10_ 1/100–1/30 = very strong evidence for H_0_, BF_10_ < 1/100 = extreme evidence for H_0_. Displayed are results of the classical linear regression analysis and the BF_10_s of the Bayesian linear regression analysis*.

People scoring higher on narcissism indicated *information validation* as a more important motive when talking about absent third parties than people scoring lower in narcissism both in work (*p* = 0.001) and private (*p* < 0.001) settings. Gathering information about others was also a more important motive for individuals scoring higher on narcissism than for those scoring lower narcissism, however, only in work settings (*p* = 0.006). Using gossip to build relationships was more often rated as relevant by individuals scoring higher on narcissism than by those scoring lower on narcissism both in work (*p* < 0.001) and private (*p* = 0.007) settings. Gossiping with a person in order to warn that person of a target person appears not to spur gossip for any of the dark personalities. Again, social enjoyment reasons are more often reported by participants scoring higher on narcissism, however, only in work-related settings (*p* = 0.05). Using gossip to negatively influence the reputation of the target person is related to narcissism and Machiavellianism. Whereas, in private settings this motive appears to be more important for individuals scoring higher on narcissism than for those scoring lower on narcissism (*p* = 0.03), in work settings it appears to be more important for individuals scoring higher on Machiavellianism than for those scoring lower on Machiavellianism (*p* = 0.006).

Bayesian regression analyses were conducted in two steps. In a first step, the BF_10_s of the models with narcissism, Machiavelliansim, and psychopathy as independent variables and the respective gossip motive as dependent variable were of interest (see Table 2; A: BF_10_). In the work setting, Bayesian linear regression analyses show that all models received support from the data with BF_10_s varying between BF_10_ = 2.30 for *social enjoyment* and BF_10_ = 122473.60 for *relationship building*. The positive BF_10_s indicate that the data are more likely under the model assuming associations between the three personality traits and the respective gossip motive than under the model assuming no association. In contrast, in the private setting the picture is not that clear-cut. The models predicting *information validation* (BF_10_ = 6.09), *relationship building* (BF_10_ = 25.36), and *negative influence* (BF_10_ = 123.43) show moderate to extreme evidence for the H_1_ indicating that the data are more likely under the model assuming associations between the three personality traits and the respective gossip motive than under the model assuming no association. The models predicting *information gathering* (BF_10_ = 0.35), *protection* (BF_10_ = 0.03), and *social enjoyment* (BF_10_ = 0.73) show anecdotal to very strong evidence for the H_0_ indicating that the data are more likely under the model that assumes no relationship than under the model including the three personality traits.

In a second step, we did not look at the models containing all three personality traits but at the models reaching the highest BF_10_ (see Table 2; B: BF_10_). For instance, when *gossip validation* in work context has been the dependent variable the model containing only narcissism reached the highest BF_10_. This indicates that the data are about 124 times more likely under the model assuming an association between narcissism and *gossip validation* than under the model assuming no such association (BF_10_ = 123.92). Taken together, the BF_10_s in the work setting showed that in 5 out of 6 analyses the BF_10_s are highest when the models include only narcissism. For instance, the data are about 492 times more likely under the model assuming *narcissism* and *information gathering* to be associated than under the null model that assumes no association (BF_10_ = 491.71). Similarly, in the private setting, in 4 out of 6 analyses the BF_10_s are highest when the model includes only narcissism.

Taken together, both classical linear regression and Bayesian linear regression show that the importance of motives mainly depends on the gossiper's narcissism.

## Discussion

### Summary

In the present study, we examined the differential importance of reasons to engage in gossip behavior. Six distinct reasons have been identified that underlie gossip behavior: *information validation, information gathering, relationship building, protection, social enjoyment*, and *negative influence*. Replicating previous research, the results show that motives were seen as differentially important (Beersma and Van Kleef, 2012). It appears that people mainly gossip for informational reasons and only marginally to harm others. This holds true in two fundamental domains of life, namely the private and the work context. In both domains the importance of motives mainly depends on the narcissism of the gossiper whereas psychopathy appears to be irrelevant for gossip motivation.

### Good or Bad?

Taken together, the results suggest that gossip is better than its reputation as people report to mainly use gossip for informational reasons and not to ruin the reputation of others. That means, when broadening the view and evaluating gossip not only with regard to social functions but also with regard the intention of the gossiper a positive impression of gossip emerges. Importantly, even individuals that are willing to dismiss commonly accepted social norms, act selfishly, and harm others for their own good appear to use gossip to tune their picture of other humans and themselves and not to harm others. Thus, even individuals with “dark” personalities rarely use gossip with a negative intention, underlining the positivity of gossip.

One might argue that this positive view on gossip arises due to data flawed by participants' tendency to socially desirable responding. However, there are several reasons challenging that argument. First, there is also evidence from observational (i.e., eavesdropping) studies showing that the content of conversation is mainly neutral in its value and only certain parts are clearly positive or negative (Levin and Arluke, 1985; Dunbar et al., 1997). And also the study by Beersma and Van Kleef (2012) shows that people mainly talk about others for informational reasons and not to negatively influence other people's reputation. Contrary to that perspective, gossip is considered as a form of passive-aggressive form of workplace bullying in work and organizational literature (e.g., Lewis and Gunn, 2007; Crothers et al., 2009; Privitera and Campbell, 2009). In line with that notion, it appears that negative gossip at the workplace is structured around “scapegoats” indicating that a large number of employees talk negatively about a small number of colleagues (Ellwardt et al., 2012a). And being the scapegoat might have disastrous effects on the individuals as, in addition, their relationship to colleagues appears to be characterized by difficulties (Ellwardt et al., 2012a). However, gossip is only one aspect of one dimension of workplace bullying (Harvey et al., 2006; Crothers et al., 2009; Privitera and Campbell, 2009). Work and organizational literature has also shown that people gossip for other than hostile intentions at the workplace (Waddington, 2005; Waddington and Fletcher, 2005; Kuo et al., 2015; see also Michelson and Mouly, 2004; Michelson et al., 2010). Moreover, research on gossip networks within the work context shows that employees more often tend to gossip positively about colleagues than negatively (Ellwardt et al., 2012a). In sum, there is work and organizational literature conceptualizing gossip outside the bullying framework but rather as a channel of informal communication and information exchange (Michelson and Mouly, 2004; Michelson et al., 2010; Kuo et al., 2015) underlining the notion that the negative reputation of gossip is not justified. Second, even people with so called “dark” personalities are not invariably triggered by malicious motives when talking about others. And individuals with “dark” personalities are not known for their desire to appear or behave socially appropriate (e.g., Foster and Trimm, 2008). Thirdly, the present study has been conducted as an online study warranting complete anonymity. Therefore, one might assume that there was no need for socially desirable responding. Thus, it appears that people, regardless of being on the “dark” side of personality or on “bright” side, mainly use gossip to tune their picture of other humans and themselves.

Considering the rather positive motives and social functions of gossip, it appears highly interesting why gossip is condemned so harshly. One might speculate that the positive social functions of gossip depend on a moderate use of gossip be it with regard to the amount or the valence of gossip. In line with that notion, research shows that individuals who show a high frequency of negative gossip are rated as highly dislikable (Farley, 2011). Also, qualitative research show that people, even though enjoying gossip, restrict themselves because they are afraid of becoming a gossip target themselves (Rodrigues et al., 2019). Assuming excessive gossip would damage trust within groups and harm individuals, one might speculate that the bad reputation of gossip restricts people from gossiping excessively. Thus, the bad reputation might also have a positive social function itself.

### Motivation, Personality, and Situation

The present results show that the reasons to talk about an absent person depend to some extent on the personality of the people being part of the gossip activity (i.e., gossiper). However, the association with personality varies between motives, traits, and situations.

#### Motives

*Validating* and *gathering information* were the most important motives in the private and the work context. Validating their view of the social world and gaining information through gossip is likely to help the individual to form a map of their social environment and their position within that social environment in the long run (Suls, 1977; Baumeister et al., 2004; Foster, 2004; De Backer et al., 2007; Sommerfeld et al., 2007; Martinescu et al., 2014). *Information validation* shows a consistent positive relationship with narcissism. The more narcissistic a person is, the more they tend to use gossip in order to validate information about others and also to gather information (at least in the work setting). As individuals scoring high on narcissism are characterized by a grandiose (and sometimes vulnerable) self-concept that causes them to search for external appreciation (Morf and Rhodewalt, 2001; Jones and Paulhus, 2011; Back et al., 2013), one might speculate that gossip is a low risk method to gain information about the self. Even though the received information makes one stack up badly against the social environment, it might be less painful because it does not happen in the “public eye”. This way, to gain social comparison information minimizes the potential psychological cost. Thus, calibrating the own perspective and gathering information about other people, and thereby about the self, appears to be important when gossiping, specifically for people who shy away from more direct forms of social comparisons due to a vulnerable self-concept.

Another reason to exchange information about a third person was to build trust to one's gossip partner. This motivation was apparent in the private setting as well as in the work setting suggesting that it might play a role in amicable relationship building as well as professional networking. Also, *relationship building* shows consistent positive associations with narcissism. The more narcissistic a person is, the more they report using gossip in order to build trust and grow closer with the gossip partner. Jonason and Schmitt (2012) claim narcissism to be the “dark” trait with “the most social core” (p. 402). In line with that notion, they were able to show that individuals scoring high on narcissism are not choosy when selecting friends. In addition, Buffardi and Campbell (2008) showed that individuals scoring high on narcissism are more active on social networking sites such as Facebook® (see also e.g., Carpenter, 2012). Jonason and Schmitt (2012) argued that being surrounded by a lot of (potential) friends is a way to satisfy the continuous need for self-validation; likewise, it may serve the need for external self-affirmation and appreciation—you need audience when you want to shine on stage (Morf and Rhodewalt, 2001). However, as more narcissistic individuals make more favorable impressions at first sight but not in the long run (Paulhus, 1998; Back et al., 2010), one might speculate that more narcissistic individuals have to keep “friend-supplies” coming. Exchanging information with others might be an easy method to form new relationships and shape the social environment according to one's needs.

The motive to warn and protect a conversational partner appears to be of similar importance as *relationship building* and *enjoyment*. It seems likely that this rather altruistic motive, on the long run, serves the function of group protection. Thus, by passing reputational information about a potential “harm-doer” to a gossip partner, the group as a whole is protected against cheaters, free-riders and alike (Dunbar, 2004b; Keltner et al., 2008; Piazza and Bering, 2008; Beersma and Van Kleef, 2011; Feinberg et al., 2012, 2014). Also, at the group level, a climate of information permeability and norm compliance is generated (e.g., Piazza and Bering, 2008; Feinberg et al., 2012). In contrast to our expectation, we did not find any negative relationship between the dark triad and the protection motive. That is, individuals scoring high on the dark triad traits gossip as often as individuals scoring low on the dark triad traits in order to protect somebody. In the light of these findings one might speculate that for “dark” individuals gossiping about cheaters and free-riders removes potential rivals and creates a climate of trust. In a climate of trust, people with dark personalities could continue to follow their self-beneficial agendas without being hindered.

Gossiping just for fun and to pass time appears to be as important as relationship building and protecting others from harm. This finding deviates from previous research where social enjoyment reasons were rated as more important than protection reasons (Beersma and Van Kleef, 2012). As in the present sample more participants are employed than in the sample of previous research (Beersma and Van Kleef, 2012), individuals are presumably more occupied and have less time for gossiping just for fun. In support of that notion, the social enjoyment motive is less important in the professional setting than in the private setting. However, this difference between work and private setting has to be interpreted with care as different *F*-test corrections come to different results and the Bayes analyses show that the data get more support when including only “motive” as predictor in the model. Curiously, specifically in a professional setting, people scoring high on narcissism tend to gossip more for social enjoyment reasons than people scoring low on narcissism. It appears as if time limits in work setting and other boundaries given by social norms in work settings do not hinder them from passing time talking about others.

In clear contrast to the bad reputation, gossiping is not mainly driven by malicious reasons. Rather, negatively influencing the reputation of others is the least important reason to gossip. As outlined earlier, this is in line with previous results from observational (i.e., eavesdropping) studies showing that the content of conversation is mainly neutral in its value and only certain parts are clearly positive or clearly negative (Levin and Arluke, 1985; Dunbar et al., 1997). Interestingly, the motivation to negatively influence the reputation of somebody else is not solely associated with narcissism but also with Machiavellianism. However, whereas individuals scoring high on narcissism appear to use gossip in a malicious way in private settings, individuals scoring high on Machiavellianism tend to bad-mouth others in professional settings. The potentially more competitive professional setting elicited more malicious gossip only by people scoring high on Machiavellianism. One might speculate that more Machiavellian individuals use gossip more strategically to gain long-term, higher order goals in domains of competition and performance (Jones and Paulhus, 2011).

Even though it appears that the reasons to gossip correspond to the social functions of gossip, we do not suppose that the different gossip motives act in the service of a single function exclusively. Rather, a single motive might serve unintentionally different social functions, presumably more than one at once. For instance, gossiping just for fun might serve a recreational function, and, at the same time, create trust and closeness facilitating relationship building. Likewise, individuals might use gossip in order to negatively influence the reputation of a target person, and, without intention, simultaneously serve the social function of group protection. To make it even more complicated, it is plausible to assume that people have different motives at the same time. Gossiping in order to protect a gossip partner might well go hand in hand with the intention to damage the reputation of the gossip target. Thus, there is much more research needed to uncover the complex interrelations between the diverse motives and social functions.

#### Personality

Interestingly, with regard to the dark triad personality traits, only narcissism shows consistent associations with motives to gossip. According to Jones and Paulhus (2011), narcissism can be distinguished from psychopathy and Machiavellianism by the type of goals they pursue. Whereas, individuals scoring high on psychopathy and Machiavellianism pursue goals of a concrete, instrumental nature, individuals scoring high on narcissism aim for goals that are of an abstract, symbolic nature (Jones and Paulhus, 2011, p. 258). Accordingly, more narcissistic individuals have a higher need for a superior identity. The identity in turn emerges in part from information provided by the social environment (Baumeister, 1997, 1998; Fiske, 2004; Jones and Paulhus, 2011). As outlined earlier, gossip appears to be a painless-and-quick mean to get information about the social surrounding and oneself; either by explicitly using gossip to validate and gather information or by trying to foster relationships in order to have an audience to act.

Other research shows that in comparison to more psychopathic and more Machiavellian individuals, more narcissistic individuals tend to use more *soft tactics* to influence others (Jonason et al., 2012). Soft tactics are designed to convince another person of the advocated behavior being in their best interest. In contrast, hard tactics are tactics which the user forces their will on another person with (Yukl and Falbe, 1990). Also, individuals scoring high on narcissism show more indirect bullying than physical bullying (Baughman et al., 2012). Assuming that gossip is a *soft tactic* or a form of indirect aggression (McAndrew, 2014), one might speculate more narcissistic individuals to be especially prone to use these methods in order to manipulate others while maintaining their social standing.

#### Situation

In the present study, the importance of gossip motives did not differ substantially between work and private situations. This might be due to similar gossip behavior across different situations or due to the fact that the distinction between work and private situations has not been precise enough. We differentiated between work and private settings assuming that these are reasonably different in terms of social norms and competitiveness. However, jobs, workplaces, and organizations are highly different in terms of normative expectations and competitiveness. Empirical research has already shown that gossip activity at the workplace depends on variables such as trust in management (Ellwardt et al., 2012c), psychological contract violations (Kuo et al., 2015), leadership (Kuo et al., 2015), ambiguity of formal communication within organizations (Crampton et al., 1998), and perceived stress and anxiety (Waddington and Fletcher, 2005). Thus, gossip activity highly depends on organizational and occupational features. Research on organizational features that hinder or facilitate workplace bullying suggest that organizations may vary on enabling structures and processes (e.g., perceived power imbalance, frustration), on motivating structures and processes (e.g., internal competition, reward system), and on precipitating processes (e.g., organizational changes; Salin, 2003; Crothers et al., 2009). As gossip is used by workplace bullies, and, therefore, considered as one aspect of workplace bullying (e.g., Lewis and Gunn, 2007; Crothers et al., 2009; Privitera and Campbell, 2009), the reasons for gossip as well as gossip frequency and valence might also depend on these organizational or occupational features. In addition, they may also depend on the years in a specific organization. The time working within an organization is probably related to the amount and intensity of relationships somebody has to others in that organization; and, therefore, might be related to the motives that steer gossip behavior. Taken together, the organizational and occupational circumstances of participants might have been so different that potential effects due to the situation might have been blurred. In addition, around 30% of our participants were students for whom private and work life are not that distinct and merge. In order to gain more knowledge about the effects of situational attributes on gossip motives, frequency, and valence future research should use experimental design or intensive longitudinal methods (i.e., diary and experience sampling).

## Limitations

One strength of the present study is that it extends previous research through the comprehensive assessment of gossip reasons. Furthermore, the present study took the challenge of assessing gossip motivation and the dark triad personality traits in a non-student sample. Additionally, we studied the importance of gossip motivations in different contexts of social life (private and work-related).

Given these strengths, some limitations need to be considered. First, the motives captured in the *Motives to Gossip Questionnaire* as well as in the extended version are based on a literature review, and, therefore, ultimately originate from the mind of researchers. Future research needs to take a more comprehensive approach and investigate whether the results can be replicated by using minds of lay persons (i.e., interviewing lay persons about their reasons). In-depth interviews might uncover additional motives to exchange information about others.

Second, considering that one focus of the study was the association between the gossip motives and the dark triad personality traits, the use of a short measure of the dark triad is questionable. The Dirty Dozen aims to capture the core aspects which are the grandiose self-view for narcissism, exploitation of others for Machiavellianism and the callousness for psychopathy (Küfner et al., 2014). Even though the Dirty Dozen Scale has shown convergent validity with comprehensive measures, using more complex measures for the respective three traits would allow deeper insight into the associations. Specifically, examining the different facets and subtypes of narcissism would facilitate the interpretation of the relationships found between narcissism and gossip motives.

Third, the order in which participants had to report on gossip in a work and in a private setting was not varied between participants. On the contrary, each participants was first asked to think about a gossip event in private setting and then in a professional setting. One might assume that thinking about a gossip event in a private setting could influence recall on gossiping in a professional context leading to similar results across situations. However, research on order effects within surveys suggests that both assimilation and contrast effects might occur (Sudman et al., 1996). For instance, in a study conducted by Schwarz and Bless (1992), participants were asked about a specific politician (i.e., Barschel) who was involved in a scandal. Those who were asked to rate the trustworthiness of politicians in general afterwards rated the trustworthiness lower than those who were asked to rate the trustworthiness of specific politicians. Thus, an assimilation occurred in the first case whereas a contrast effect emerged in the second case. As in the present study the evaluation of two specific events were required, a contrast effect would have been more likely to occur (see also Schwarz and Bless, 1991). However, as our data shows no differences between these two events, it is reasonable to assume that no order effects emerged at all. Another argument speaking against the assumption that an assimilation effect has occurred is the operation of conversational norms that prohibit redundancy. More specifically, respondents may deliberately ignore information that has already been provided in response to previous question (Schwarz and Bless, 1991; Schwarz et al., 1991; Schwarz, 1999). Thus, when thinking about the second gossip event participants might have explicitly thought about a gossip event differing from the previous one. Nevertheless, future research needs to find a concluding answer.

Fourth, in the *Motives to Gossip Questionnaire* participants are asked to think about their reasons to gossip in a specific situation capturing their *motivation* to gossip in that situation. This approach might harbor potential threats. Participants have to consult their autobiographical memory to identify relevant behavioral events, and, in addition, remember in detail the reasons for this behavior (e.g., Sudman et al., 1996). Remembering an event depends in part on the depth and elaboration of the encoding process of the event. The depth and elaboration of the encoding reflects variables such as distinctiveness, emotional impact, and duration. Events that are unusual, dramatic, or lasting ensure that a rich representation is formed and are stored in the long-term memory. Thus, unusual or emotional arousing gossip events might be remembered easily whereas irregular but frequent and relatively unimportant gossip events are not retrieved easily and perhaps forgotten entirely (Sudman et al., 1996; Tourangeau, 2000). Consequently, participants might have selected highly salient examples of gossip events that are not representative of more frequent gossip sessions. However, at the same time the rich representations of these unusual events of gossip make it more plausible that participants have a vivid and detailed memory and can therefore recall related aspects such as motivations for behavior more easily (Tourangeau, 2000). An alternative method might be to ask participants to provide information about their tendency to gossip for certain reasons across different situations and across time. Such a procedure would presumably detach the dependency of single highly salient gossip events and would also open the opportunity to study individual differences in stable underlying motives.

Fifth, because of the relatively small sample size of *N* = 134 the power of the analysis might have been reduced resulting in a higher probability of type-II error. And indeed, a post hoc power analysis for the repeated ANOVA revealed that the *F*-test of the main effect “situation” did not achieve sufficient power (0.40) to detect an effect. Similarly, the test of the interaction effect did not achieve sufficient power (0.68). Also, *post-hoc* power analysis for the two regression analyses not reaching conventional levels of significance (i.e., for *information gathering* and *protection* in private setting) revealed that the *F*-tests did not achieve sufficient power (0.13 for *protection* and 0.58 for *information gathering*). To address the issue of sample size, the analyses were repeated using Bayesian statistics (e.g., van de Schoot et al., 2013). Mirroring the results of the classical repeated ANOVA, the Bayesian repeated ANOVA revealed that the “situation” as well as the interaction were not meaningful. Also, the Bayesian linear regression mirrors the results of the classical linear regression analyses. Thus, both kinds of analyses draw a very similar picture of the results emphasizing their reliability.

Finally, in the present paper, concurrent associations between personality and reasons to gossip were studied. However, to fully understand the complex interplay between personality, gossip behavior and long term effects of gossip (i.e., social functions such as facilitation of relationship building, protection, facilitation of social learning) longitudinal studies are needed.

## Conclusion

Gossip runs like a thread through our social world. Regardless of important social functions, gossip has a rather negative reputation. The present study shows that the negative reputation is not justified as individuals indicate they mainly use gossip for informational reasons and not to harm others. And, even though the motives to gossip depend on the gossiper's personality (i.e., dark triad personality), also individuals with “dark” personalities appear to use gossip to tune their picture of other humans and themselves.

## Ethics Statement

The study conforms with the Declaration of Helsinki and the ethics guidelines of the German Psychological Society. In accordance with the national and institutional guidelines, ethical approval was not required for this study.

## Author Contributions

F-MH, CK, and MP contributed conception and design of the study and performed the statistical analysis. CK and MP organized the database. F-MH wrote the first draft of the manuscript. All authors contributed to manuscript revision, read and approved the submitted version.

### Conflict of Interest Statement

The authors declare that the research was conducted in the absence of any commercial or financial relationships that could be construed as a potential conflict of interest.

## Appendix

**The German Version of the Revised Motives to Gossip Questionnaire**

In unserem **privaten** Alltag kommt es häufig vor, dass wir über eine dritte Person sprechen, die nicht anwesend ist. Denken Sie an eine vergangene Situation in Ihrem **privaten** Alltag, in der Sie Teil eines solchen Gesprächs (über eine abwesende Person) waren. Halten Sie sich diese Situation im Folgenden vor Augen und denken Sie an Gründe, die Sie für das Gespräch hatten. Nehmen Sie dementsprechend Stellung zu folgenden Aussagen:

Ich habe an dieser Unterhaltung aus folgenden Gründen teilgenommen …

IV  …um herauszufinden ob die Person, mit der ich gesprochen habe, genauso über die abwesende Person denkt.
IV  …um unsere Gedanken über die abwesende Person zu vergleichen.
IV  …um herauszufinden, ob die Person, mit der ich gesprochen habe, meiner Meinung ist.
IG  …um Informationen über die abwesende Person zu sammeln.
IG  …um Neuigkeiten über die abwesende Person in Erfahrung zu bringen.
IG  …um Auskünfte über die abwesende Person einzuholen.
RB  …um die Beziehung zu der Person, mit der ich geredet habe, zu vertiefen.
RB  …um das Vertrauen der Person, mit der ich geredet habe, zu gewinnen.
RB  …um mich mit der Person, mit der ich geredet habe, gut zu stellen.
P     …um die Person, mit der ich gesprochen habe, vor der abwesenden Person zu schützen.
P     …um die Person, mit der ich gesprochen habe, davor zu schützen von der abwesenden Person ausgenutzt zu werden.
P     …um die Person, mit der ich gesprochen habe, vor dem Verhalten der abwesenden Person zu warnen.
SE  …zum Vergnügen.
SE  …weil es mir Spaß bereitet hat.
SE  …weil wir uns die Zeit vertreiben wollten.
NI  …um die abwesende Person in einem schlechten Licht darzustellen.
NI  …um schlecht über die abwesende Person zu sprechen.
NI  …um den Ruf der abwesenden Person zu schädigen.

Respondents indicate the degree to which they agree on a 7-point scale (1 = trifft überhaupt nicht zu, 7 = trifft voll zu). For the work-related situation the term “private” (“privat”) was replaced with “work-related” (“beruflich”).
